# P-2025. Resistance Analyses from the Remdesivir Phase 2/3 CARAVAN Study in Pediatric and Neonatal Participants Hospitalized with COVID-19

**DOI:** 10.1093/ofid/ofae631.2181

**Published:** 2025-01-29

**Authors:** Jasmine Moshiri, Jiani Li, Lauren Rodriguez, Dong Han, Simin Xu, Pui Yan Ho, Nadine Peinovich, Clarissa Martinez, Silvia Chang, Kathryn Kersey, Jason K Perry, Danielle P Porter, Charlotte Hedskog

**Affiliations:** Gilead Sciences, Inc., Foster City, California; Gilead Sciences, Inc., Foster City, California; Gilead Sciences, Inc., Foster City, California; Gilead Sciences, Inc., Foster City, California; Gilead Sciences, Inc., Foster City, California; Gilead Sciences, Inc., Foster City, California; Gilead Sciences, Inc., Foster City, California; Gilead Sciences, Inc., Foster City, California; Gilead Sciences, Foster City, California; Gilead Sciences, Inc., Foster City, California; Gilead Sciences, Inc., Foster City, California; Gilead Sciences Inc, Foster City, California; Gilead Sciences, Inc., Foster City, California

## Abstract

**Background:**

Remdesivir (RDV) is a nucleotide analog prodrug approved for treatment of COVID-19. Here we present comprehensive SARS-CoV-2 resistance analyses from all cohorts of the Phase 2/3 CARAVAN study, which demonstrated the safety of RDV for treatment of COVID-19 in pediatric participants from birth to < 18 years of age.

**Figure:**
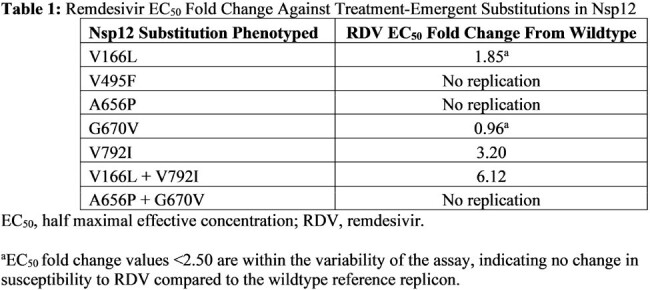

**Methods:**

CARAVAN was a single-arm, open-label study wherein pediatric participants received IV RDV for ≤10 days. Full-genome deep sequencing of SARS-CoV-2 was performed on respiratory samples collected on Days 1 (baseline), 3, 5, 7, and/or 10. In vitro antiviral activity of RDV against substitutions observed in the SARS-CoV-2 RNA replication complex was evaluated using a subgenomic replicon system. Substitutions were phenotyped if they met any of the criteria: 1) baseline Nsp12 substitutions detected in ≥3 participants; 2) baseline replication complex substitutions detected in ≥2 participants with viral RNA increase postbaseline; 3) treatment-emergent postbaseline replication complex substitutions.

**Results:**

Of 58 participants enrolled and treated, baseline sequencing data were obtained from 37 participants. Six baseline amino acid polymorphisms met criteria for phenotyping, none of which impacted RDV susceptibility (EC_50_ fold change ≤1.55). Baseline and postbaseline sequencing data were obtained from 28 participants. Five treatment-emergent substitutions in Nsp12 were identified in 3 participants as mixtures with wildtype. Of these, only Nsp12 V792I alone and in combination with V166L showed low-level reduced susceptibility to RDV (Table 1). The participant in whom V166L and V792I were observed achieved clinical recovery and was released from the hospital on Day 13. Structural modeling suggests V166L and V792I are located on or adjacent to motif D of the viral RNA-dependent RNA polymerase and may alter the dynamics of nucleoside triphosphate incorporation. Six treatment-emergent substitutions in Nsp9, Nsp10, and Nsp13 were identified in 3 participants; none impacted RDV susceptibility (fold change ≤0.74).

**Conclusion:**

The results of resistance analyses from the CARAVAN study support a high barrier to clinically meaningful resistance to RDV in pediatric patients with COVID-19.

**Disclosures:**

Jasmine Moshiri, PhD, Gilead Sciences, Inc.: Employee|Gilead Sciences, Inc.: Stocks/Bonds (Public Company) Jiani Li, PhD, Gilead Sciences, Inc.: Employee|Gilead Sciences, Inc.: Stocks/Bonds (Public Company) Lauren Rodriguez, PhD, Gilead Sciences, Inc.: Employee|Gilead Sciences, Inc.: Stocks/Bonds (Public Company) Dong Han, MS, Gilead Sciences, Inc.: Employee|Gilead Sciences, Inc.: Stocks/Bonds (Public Company) Simin Xu, MS, Gilead Sciences, Inc.: Employee|Gilead Sciences, Inc.: Stocks/Bonds (Public Company) Pui Yan Ho, PhD, Gilead Sciences, Inc.: Employee|Gilead Sciences, Inc.: Stocks/Bonds (Public Company) Nadine Peinovich, MPH, Gilead Sciences, Inc.: Employee|Gilead Sciences, Inc.: Stocks/Bonds (Public Company) Clarissa Martinez, MPH, Gilead Sciences, Inc.: Employee|Gilead Sciences, Inc.: Stocks/Bonds (Public Company) Silvia Chang, Masters, Gilead Sciences, Inc.: Employee|Gilead Sciences, Inc.: Stocks/Bonds (Public Company) Kathryn Kersey, MS, Gilead Sciences, Inc.: Employee|Gilead Sciences, Inc.: Stocks/Bonds (Public Company) Jason K. Perry, PhD, Gilead Sciences, Inc.: Employee|Gilead Sciences, Inc.: Stocks/Bonds (Public Company) Danielle P. Porter, PhD, Gilead Sciences, Inc.: Employee|Gilead Sciences, Inc.: Stocks/Bonds (Public Company) Charlotte Hedskog, PhD, Gilead Sciences, Inc.: Employee|Gilead Sciences, Inc.: Stocks/Bonds (Public Company)

